# Electronic control of H^+^ current in a bioprotonic device with Gramicidin A and Alamethicin

**DOI:** 10.1038/ncomms12981

**Published:** 2016-10-07

**Authors:** Zahra Hemmatian, Scott Keene, Erik Josberger, Takeo Miyake, Carina Arboleda, Jessica Soto-Rodríguez, François Baneyx, Marco Rolandi

**Affiliations:** 1 Department of Electrical Engineering, University of California Santa Cruz, Santa Cruz, California 95064, USA; 2 Department of Materials Science and Engineering, University of Washington, Seattle, Washington 98195, USA; 3 Department of Electrical Engineering, University of Washington, Seattle, Washington 98195, USA; 4 Department of Chemical Engineering, University of Washington, Seattle, Washington 98195, USA

## Abstract

In biological systems, intercellular communication is mediated by membrane proteins and ion channels that regulate traffic of ions and small molecules across cell membranes. A bioelectronic device with ion channels that control ionic flow across a supported lipid bilayer (SLB) should therefore be ideal for interfacing with biological systems. Here, we demonstrate a biotic–abiotic bioprotonic device with Pd contacts that regulates proton (H^+^) flow across an SLB incorporating the ion channels Gramicidin A (gA) and Alamethicin (ALM). We model the device characteristics using the Goldman–Hodgkin–Katz (GHK) solution to the Nernst–Planck equation for transport across the membrane. We derive the permeability for an SLB integrating gA and ALM and demonstrate pH control as a function of applied voltage and membrane permeability. This work opens the door to integrating more complex H^+^ channels at the Pd contact interface to produce responsive biotic–abiotic devices with increased functionality.

## Abstract (web summary)

Conventional electronics use electrons as charge carriers whereas biological systems use ions, confounding integration of electronics with biology. Here the authors show voltage-regulated flow of protons across a supported lipid bilayer using the ion channel Gramicidin A and the voltage-gated ion channel Alamethicin.

## Graphical abstract

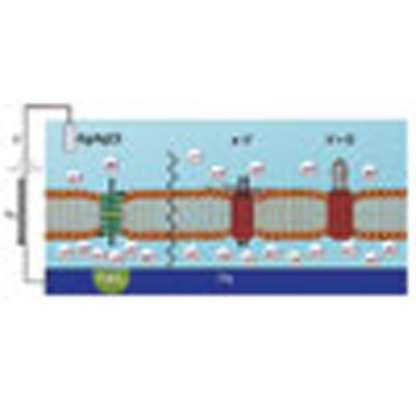

From electroceuticals^1^ to wearable devices^2^, and from electronic plants^3^ to edible electronics^4^, interfacing electronic devices with biological systems promises new therapies and device functionalities beyond silicon^5^,^6^. In biological systems, most of the communication between cells is mediated by membrane proteins and ion channels that passively allow or actively control the flow of ions and small molecules across the cell membrane^7^. In this fashion, complex functions such as muscle contraction, neuronal signalling and metabolism are achieved. Membrane proteins are studied using patch clamps^8^, micropore arrays^9^ and electrode-supported lipid bilayers^10^,^11^,^12^,^13^, and passive transmembrane ionic transport is controlled by local electrical and chemical potential gradients according to the Nernst–Planck equation^14^,^15^. While most common ions are Na^+^, K^+^ and Cl^−^, proton (H^+^) currents and concentration [*H*^*+*^] gradients play important physiological roles^16^. Examples include oxidative phosphorylation in mitochondria^17^, light-activated H^+^ pumping by archaeal bacteriorhodopsins^18^, H^+^-activated bioluminescence in dinoflagellates^19^, flagellar propulsion in bacteria^20^, voltage gated H^+^ channels^21^ and antibiotic action by polypeptides such as Gramicidin^22^.

Ionic currents and membrane proteins are the most intimate interface for electronics to communicate with cells and biological systems^23^. However, conventional electronics typically use electrons as charge carriers instead of ions. To address this issue, gramicidin and bacteriorhodopsin have been integrated as gating elements with carbon nanotubes^24^, silicon nanowires^25^ and organic field effect transistors^26^ to develop biosensors with increased functionality. Organic polymers^27^,^28^,^29^ with ionic^30^,^31^ and mixed conductivity have been used to record and stimulate physiological functions and even assembled into logic circuits^32^. We have recently demonstrated control of H^+^ flow in bioprotonic field effect transistors (H^+^-FETs)^33^,^34^,^35^ and memories^36^, and integrated these devices with enzymatic logic gates^37^. H^+^-FETs built with squid reflectin proteins have also been described^38^,^39^,^40^. At the heart of these devices is the use of the Pd/PdH_*x*_ couple as a contact that enables the translation of an H^+^ current into an electronic current and thus serves as a transducer between biological systems and electronics^35^,^41^. Here, we fabricate and characterize bioprotonic devices incorporating ion channels and Pd/PdH_*x*_ contacts to control H^+^ currents and modulate pH gradients across phospholipid membranes (Fig. 1). These devices comprise a supported lipid bilayer (SLB) that mimics the function of a cell membrane at the Pd/solution interface and acts as a self-sealing support for the insertion of the ion channels Gramicidin A (gA) and Alamethicin (ALM). We show that gA can be used to linearly control H^+^ currents as function of voltage while ALM functions as a voltage-gated channel analogous to an ON-OFF switch. This is a unique and novel architecture compared to prior work with electron conducting Au^42^ and Pt^43^ electrodes that allows for direct interfacing of H^+^ current from the ion channels.

## Results

### Device architecture

Pd contacts are evaporated in areas ranging from 10 μm^2^ (2 × 5 μm) to 200 μm^2^ (2 × 100 μm) onto a Si wafer with a 100 nm SiO_2_ insulating layer. Microfluidic channels are defined by lithographically patterned SU-8 photoresist and sealed with a polydimethylsiloxane layer comprising of inlet and outlet ports (Supplementary Fig. 1). The channels are filled with a standard buffered solution (5 mM PBS and 100 mM KCl, pH=7.0) and an Ag/AgCl electrode is inserted into the solution to serve both as a reference electrode and as a counter electrode in a two terminal setup.

**Figure 1:**
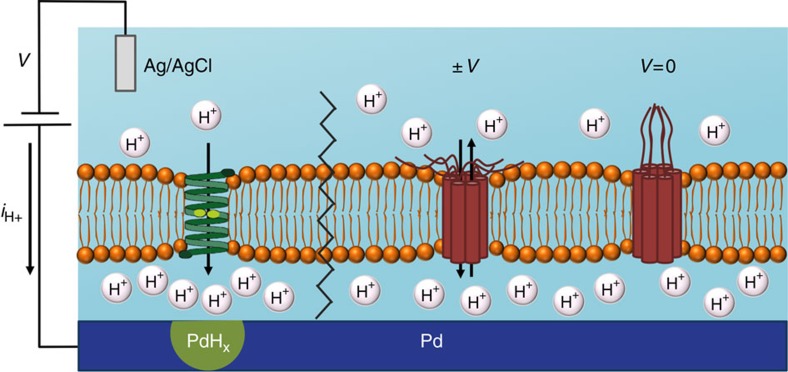
Schematic depiction of the ion channel bioprotonic device. (Left) A bioprotonic device with integrated Gramicidin (gA) supports the flow of H^+^ across the SLB upon application of a negative voltage (*−V*) to the Pd contact. When H^+^ reach the surface of the Pd contact, they are reduced to H by an incoming electron and diffuse into Pd to form a hydride (PdH_*x*_). A reduction current at the Pd contact is measured as current density (−*i*_H+_). (Right) In a bioprotonic device with integrated Alamethicin (ALM), applying a negative or positive voltage (V) above a threshold value at the Pd contact opens the gate and allows H^+^ flow across the SLB, turning the device ON. At *V*=0 mV, no H^+^ flows across ALM and the device switches OFF.

We apply a potential difference between the Pd contact and the Ag/AgCl electrode (*V*) and measure the resulting H/H^+^ current density (*i*_H+_) where oxidation of H corresponds to a positive *i*_H+_ and reduction of H^+^ corresponds to a negative *i*_H+_ (Fig. 1). For −*V* applied to the Pd contact, electrons flow from the Pd contact and reduce H^+^ to H at the Pd/solution interface. H then absorbs into the Pd to form PdH_*x*_ with *x* up to 0.6. Conversely, for +*V*, H oxidizes into H^+^ at the Pd/solution interface, and is released into solution. This results in the collection of electrons by the Pd contact, which in turn yields a positive *i*_H+_ value. In short, by measuring *i*_H+_, we effectively monitor the flow of H^+^ between the solution and the Pd contact mediated by H^+^+e^−^⇔H, and subsequent absorption of H into Pd to form PdH_*x*_ or desorption of H from the contact to form H^+^ (refs 37, 44).

To electrically isolate the Pd contact from the solution and provide a template for ion channel insertion, we deposit a 1,2-Dioleoyl-sn-glycero-3-phosphocholine (DOPC, Sigma-Aldrich Lipids) SLB onto the Pd contact using vesicle fusion^45^. Force rupture measurements by Atomic Force Microscopy (AFM)^46^ show that the thickness of the SLB membrane is 4.8±0.7 nm (*n*=20, for a device with dimension of 2 × 50 μm), which is close to the expected value of 5.5 nm for a DOPC bilayer^47^,^48^ (Supplementary Fig. 2 and Supplementary Note 1). In essence, the SLB mimics a cell membrane that electrically insulates the Pd contact (*ρ*∼3 G Ω cm^−1^; see Supplementary Fig. 3 and Supplementary Table 1) and divides the solution into two volumes. We refer to the larger volume containing the Ag/AgCl electrode as the bulk solution (B). We refer the small volume between the SLB and the Pd contact, as the isolation layer (IL). The IL provides lubrication and mobility to the SLB^49^,^50^,^51^,^52^, creating an excellent platform for the investigation of cellular processes involving small size molecules such as gA and ALM.

### Voltage control of H^+^ flow with Gramicidin A

**Figure 2:**
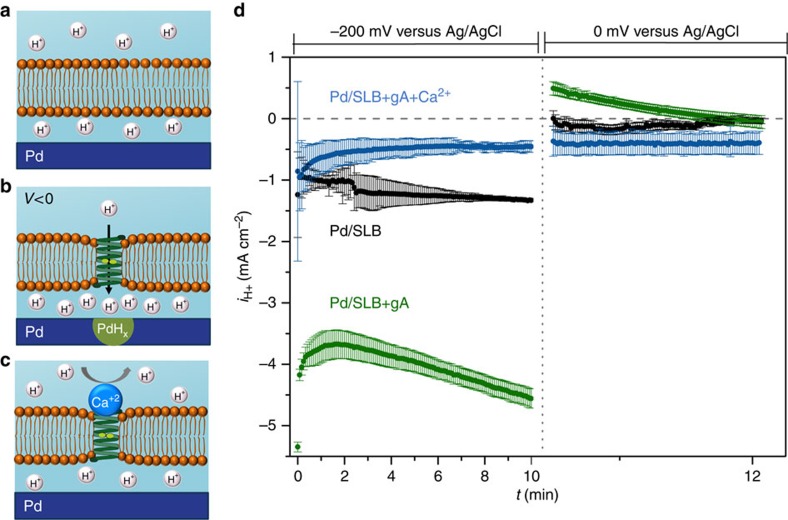
Schematics of bioprotonic gA devices. (**a**) Pd contact coated with a SLB. The SLB inhibits the flux of H^+^ from the bulk solution to the Pd/solution interface. (**b**) Pd contact with SLB incorporating gA is semipermeable to H^+^, with gA channels facilitating the rapid flow of H^+^ to the Pd/solution interface. (**c**) Addition of 1 mM Ca^2+^ to the bulk solution, blocks gA and prevents the flow of H^+^ to the Pd/solution interface. (**d**) *i*_H+_ versus time plot for *V*=−200 mV and *V*=0 mV. Black trace SLB, green trace SLB+gA, blue trace SLB+gA blocked by Ca^2+^. (The data are collected from 3 different devices with different dimensions: Pd / SLB: 3 different devices of 2 × 50 μm, Pd/SLB+gA: 2 × 20 μm, 2 × 50 μm, 2 × 70 μm, Pd/SLB+gA+Ca^2+^: 2 different devices of 2 × 50 μm and 1 device of 2 × 70 μm. The error bars are the root mean square of the displacement of the data from the average value).

Gramicidin A (gA) is a short helical polypeptide from *Bacillus brevis* that dimerizes in lipid bilayers to form a transmembrane channel that allows the passage of small cations (including H^+^) while remaining impermeable to anions^53^. To control the flow of H^+^ as a function of *V*, we integrate gA in the SLB of our devices. In the absence of gA and at an applied *V*=−200 mV, we measure *i*_H+_=−1.2 mA cm^−2^ (Fig. 2a,d). This small *i*_H+_ indicates that few H^+^ diffuse across the bilayer to become reduced at the Pd surface. To confirm this result, we set *V*=0 mV after applying *V*=−200 mV for 10 min. If any H^+^ flow were to occur across the SLB during the *V*=−200 mV step, a significant amount of PdH_*x*_ should form at the Pd/solution interface^37^. This PdH_*x*_ has a higher protochemical potential (*μ*_H+_) than the solution of pH=7.0 at *V*=0 mV, leading to H oxidation at the PdH_*x*_ contact, H^+^ flow from the PdH_*x*_ into the IL, and giving rise to a measurable positive *i*_H+_ (ref. 34). The black trace in Fig. 2d (right hand side panel) shows that this is clearly not the case.

In contrast, the bioprotonic device with gA and subjected to *V*=−200 mV experiences a large *i*_H+_ that continually increases to a maximum of *i*_H+_=−4.5 mA cm^−2^ at *t*=10 min (Fig. 2b,d), because the gA inserted within the SLB create pathways for H^+^ flow. The dependence of *i*_*H+*_ as a function of time will be discussed in the modelling section. Setting *V*=0 mV after 10 min causes H^+^ to transfer from the PdH_*x*_ to the solution with a maximum *i*_H+_=0.5 mA cm^−2^ (Fig. 2d, green trace). The presence of a large oxidation peak for H^+^ at 50 mV in the *I*–*V* sweep confirms that this transfer is indeed occurring (Supplementary Fig. 4). To verify that gA was responsible for H^+^ flow across the SLB, we added 1 mM Ca^+2^ to block H^+^ transfer across the gA channel (Fig. 2c)^25^. Under these conditions, for *V*=−200 mV we measure *i*_H+_=−0.7 mA cm^−2^ and subsequently measure *i*_H+_=−0.5 mA cm^−2^ when setting *V*=0 mV after 10 min (Fig. 2d, blue trace). These values of *i*_H+_ are comparable to those measured when no gA is incorporated in SLB and are consistent with Ca^2+^ blocking gA channels.

### Alamethicin voltage-gated H^+^ switches

**Figure 3:**
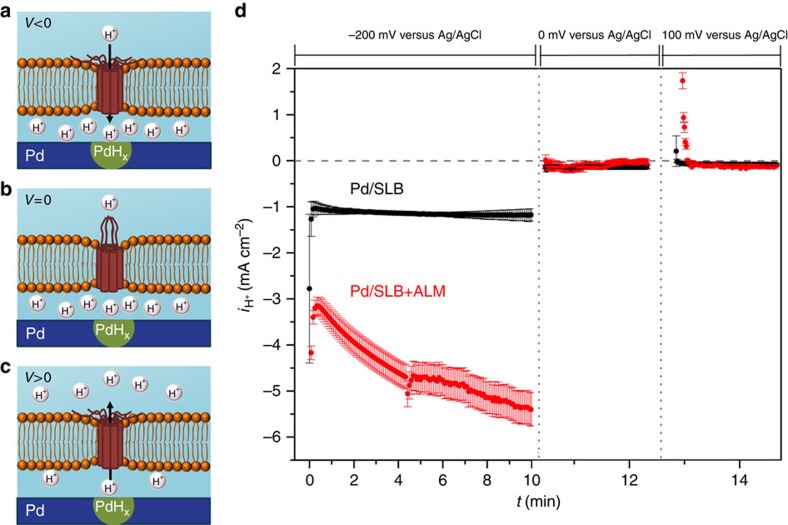
Schematics of bioprotonic voltage-gated devices. (**a**) Pd contact with SLB and ALM. At *V*=−200 mV, ALM channels open and H^+^ flows to the Pd/solution interface. (**b**) PdH_*x*_ contact with SLB and ALM remains closed under *V*=0 mV and there is no H^+^ flow. (**c**) PdH_*x*_ contact coated with SLB and ALM can only oxidize H under a small positive voltage of *V*=100 mV which is also required to open ALM channels and allow for flow of H^+^ from the PdH_*x*_ contact to bulk solution. (**d**) *i*_H+_versus time plot for *V*=−200, 0 and 100 mV, SLB black trace and SLB integrating ALM red trace. (The data is collected from 3 different devices with the different dimensions, Pd/SLB: 3 different devices of 2 × 50 μm, Pd/SLB+ALM: 2 × 20 μm, 2 × 40 μm, 2 × 50 μm. The error bars are the root mean square of the displacement of the data from the average value).

To demonstrate that our device architecture can be used to create a voltage-dependent switch that turns the H^+^ flow on and off between bulk solution and isolation layer (Fig. 3), we integrate alamethicin (ALM) into the SLB. Alamethicin is a 20-amino-acids long peptide from the fungus *Trichoderma viride*. ALM undergoes spontaneous insertion into lipid bilayers and forms a voltage-gated channel when 4-to-6 molecules associate to form an α-helical bundle^54^,^55^,^56^. An asymmetric threshold voltage above ∼60 mV induces reorientation of the helices, thereby opening the channel to selective transport of cations, including H^+^, in the direction of *V* (ref. 57). For *V* values inferior to this threshold, ALM is closed and there is no H^+^ flow across the membrane in spite of differences in [*H*^*+*^], which typically creates a driving force for H^+^ flow (Fig. 3).

Similar to the gA device, we measure *i*_H+_=−5.5 mA cm^−2^ at *V*=−200 mV for an ALM integrated bioprotonic device; at *V*=−200 mV the ALM channel is open, allowing flow of H^+^ (Fig. 3a,d). When the voltage is set at *V*=0 mV, *i*_H+_=0 mA cm^−2^. This is because the ALM channel is closed, and no H^+^ flow occurs between IL and B, even after PdH_*x*_ formation. For *V*=100 mV, the ALM channel opens and we measure *i*_H+_=1.9 mA cm^−2^, indicating that H^+^ flows from the PdH_*x*_ contact and across the SLB. As a control, we added 40 mM urea to the bulk solution to irreversibly disrupt the ALM channel structure and stop H^+^ flow across the SLB^58^. Under these conditions, we find that *i*_H+_=−1.2 mA cm^−2^ for *V*=−200 mV and *i*_H+_=0 mA cm^−2^ for both *V*=0 mV and *V*=100 mV (Supplementary Fig. 5), confirming that ALM is responsible for the bioprotonic device behaviour. Moreover, as is the case with the gA device, the H^+^ oxidation peak at 50 mV dominates the *I*–*V* sweep. However, for *V*>0 mV, *i*_H+_ in the ALM device has a rectifying diode-like behaviour (Supplementary Fig. 6). We conclude that the voltage-gated functionality of the ALM channel is maintained on its integration into the device's SLB.

To summarize, the device switches ON when ALM voltage-gated channels are open, allowing H^+^ flow across the membrane and in the direction of *V*. The device is OFF when *V*=0 mV, a voltage below the threshold value needed for ALM opening. Thus, an ALM device can be used to modulate H^+^ flow and [*H*^*+*^] between IL and B, much like cells control [*H*^*+*^] differences between cytosol and extracellular space.

### A model for H^+^ transfer across gA and ALM channels

**Figure 4:**
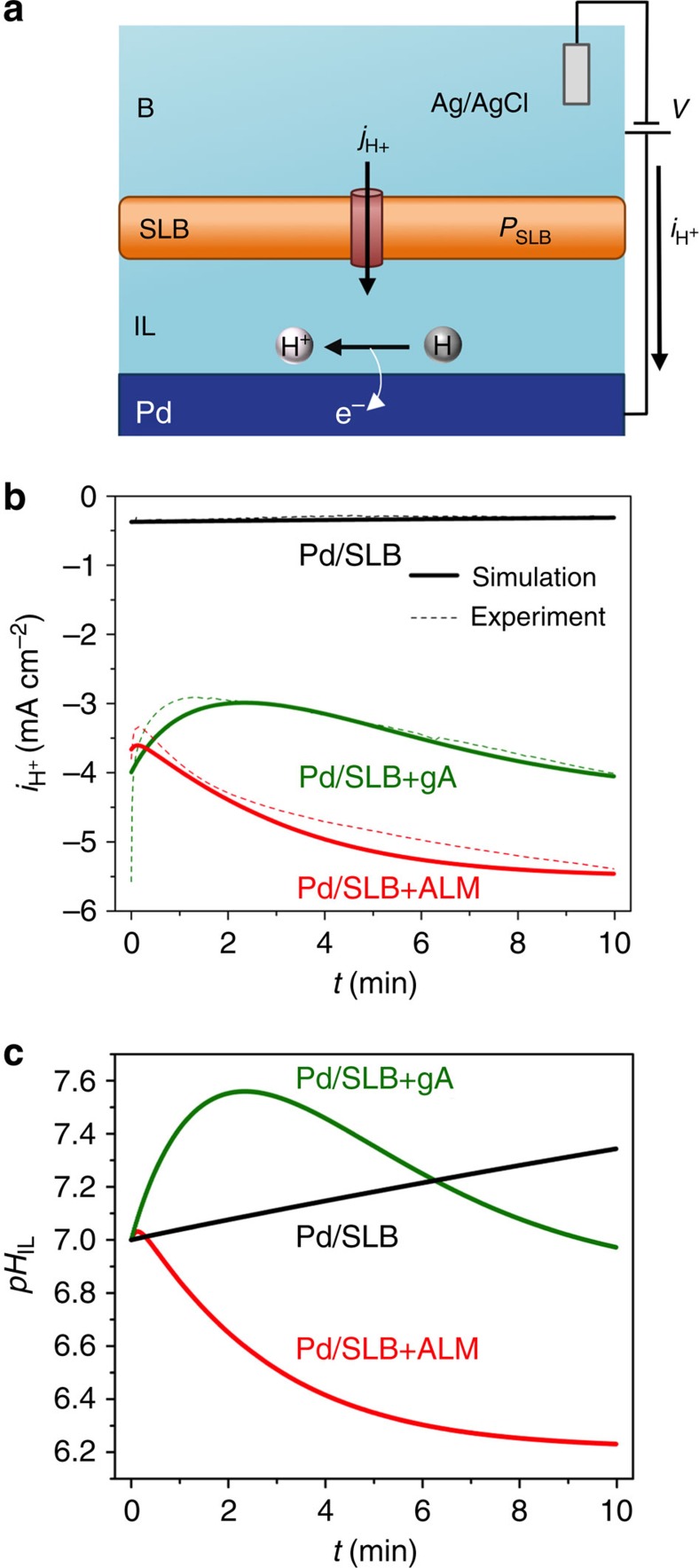
Analytical description of channel permeability. (**a**) Schematic of membrane modelling parameters. (B, bulk solution; SLB, Supported Lipid Bilayer membrane; IL, isolation layer; *P*_SLB_, SLB permeability; [*H*^*+*^]_IL_, H^+^ concentration in isolation layer; *j*_H+_, H^+^ flux across the membrane; *i*_H+_, H^+^ redox current density; *V*=−200 mV; *j*_H+_ and *i*_H+_ are computed with equations 2 and 3, respectively. A rise in [*H*^*+*^]_IL_ increases the d[*H*^*+*^] between Ag/AgCl electrode and Pd contact for H^+^ reduction, according to the Nernst equation (equation 4). (**b**) Iterative simulation of *i*_H+_ at the Pd/solution interface for SLB (black trace), SLB incorporating gA (green trace), and SLB incorporating ALM (red trace) bioprotonic device. A combination of GHK and Tafel equations was used to describe the behaviours of membrane and Pd contact. (**c**) Change in *pH*_IL_ using the parameters from the simulation.

To better understand the dynamics of H^+^ flow in our biotic–abiotic devices, we modelled H^+^ transport characteristics using the Nernst–Plank equation to fit our device current density data, *i*_H+_ (Fig. 4). To this end, we divide the device into four distinct layers: the bulk solution (B); a Supported Lipid Bilayer membrane (SLB) with variable permeability (*P*_SLB_) that encompasses the SLB with integrated gA or ALM; the isolation layer (IL); and the Pd/PdH_*x*_ contact (Fig. 4a). When a negative voltage *−V* is applied to the Pd contact, H^+^ flow from B across the SLB and into the IL. We quantify this transport as the H^+^ current density across membrane, *j*_H+_. A portion of the H^+^ from *j*_H+_ contribute to an increase in [*H*^*+*^] in the IL, [*H*^*+*^]_IL_, while the remaining H^+^ are reduced to H at the Pd contact and form PdH_*x*_. We assume that *i*_H+_ measured between the Pd contact and the Ag/AgCl electrode arises entirely from electrons participating in H^+^ oxidation (positive *i*_H+_) or reduction processes (negative *i*_H+_), and we quantify it as current density, *i*_H+_.

From conservation of mass (equation 1)^59^


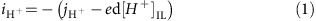


where, *e* is elementary charge=1.6 × 10^−19^ C, and d[*H*^*+*^]_IL_ is the incremental change in molar concentration of H^+^ in the IL in moles m^−3^. *j*_H+_ is obtained by inserting the Goldman–Hodgkin–Katz (GHK) solution^60^ into the Nernst–Planck equation (equation 2)^59^:


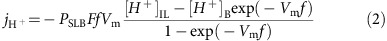


where *P*_SLB_ is the permeability of H^+^ through the SLB, *F* is Faraday's constant, *f* is F/RT, or 38.66 V^−1^, *V*_m_ is the potential difference across the membrane and [*H*^*+*^]_B_ is the H^+^ concentration in the bulk layer in moles m^−3^ (10^−4^ mol m^−3^). We set *V*_m_=*V* and assume that the potential drop across B is negligible relative to the potential drop across the membrane. When no gA or ALM channels are present, the resistance of the SLB, *R*_SLB_≈GΩ (Figs 2 and 3), is six orders of magnitude higher than the resistance of B (*R*_B_=0.6 kΩ).

We derive *i*_H+_ using a modified version of the Tafel equation^61^ (equation 3):


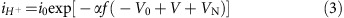


where *i*_0_ is the Pd exchange current density (a constant describing forward and reverse *i*_H+_ at the Pd/solution interface when they are equal to each other and the net *i*_H+_ is 0 mA cm^−2^), *α* is the transfer coefficient, *V*_0_ is the redox potential between Pd and Ag/AgCl (−0.22233 V), and *V*_N_ is the Nernstian potential created by the change in [*H*^*+*^] across the SLB. *V*_N_ is derived from (equation 4):


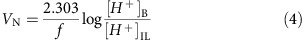


We assume that [*H*^*+*^]_B_ remains constant and calculate the change in [*H*^*+*^]_IL_ from the change of pH in the IL, d*pH*_IL_, assuming a buffering capacity for the solution, *β* (Supplementary Note 2)^62^:


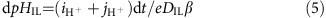


where *D*_IL_ is the thickness of the isolation layer. The resulting d[*H*^*+*^]_IL_ is (ref. 59):


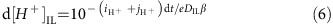


To fit *i*_H+_ to the experimental data, we programed an iterative solution to the above model using the membrane permeability, *P*_M_, as a fitting parameter. For *V*=−200 mV and *D*_IL_=0.5 nm the model fits the experimental data well using membrane permeabilities of *P*_SLB_=0.006 s^−1^ (unmodified SLB), *P*_SLB+gA_=0.58 s^−1^ (SLB with integrated gA) and *P*_SLB+ALM_=0.74 s^−1^ (SLB with integrated ALM). Consistent with our expectation, insertion of either gA or ALM into the SLB increases membrane permeabilities by three orders of magnitude. We did not control or accurately measure the density of the channels and these permeabilities are characteristic of the device and do not represent the absolute conductivities of individual gA or ALM.

We next calculate the change in *pH*_IL_ using the above permeabilities (Fig. 4c). Qualitatively, if the rate at which H^+^ are reduced at the Pd contact is higher than the rate at which H^+^ permeate the membrane (|*i*_H+_|>|*j*_H+_|), *pH*_IL_ will increase with respect to the *pH*_B_. Considering protochemical potential differences among B, IL and the Pd/PdH_*x*_ contact, an acidic *pH*_IL_ will favour transfer of H^+^ from the IL into Pd (|*i*_H+_|>|*j*_H+_|), while a basic *pH*_IL_ will favour transfer of H^+^ from B to the IL through the gA and ALM channels. For a naked SLB, our model predicts that some H^+^ reduction will occur at the Pd surface. Very few H^+^ leak through the membrane and *pH*_IL_ increases slowly (Fig. 4c, black trace). For an SLB integrating gA, the model predicts an initial increase in *pH*_IL_ followed by a progressive decrease as more H^+^ flow through the gA channel over time. These results can be rationalized as follows: at the initial *pH*_IL_=7, reduction at the Pd contact interface (|*i*_H+_|) is favoured with respect to flow through gA channels (|*j*_H+_|) because *pH*_B_=*pH*_IL_ and the only driving force for flow along the gA channel is *V*. As *pH*_IL_ increases, |*j*_H+_| increases and |*i*_H+_| decreases (Fig. 4b) due to the [*H*^*+*^] difference between B and IL driving H^+^ flow across gA. In the case of an SLB integrating ALM, the dynamics for *i*_H+_ and *pH*_IL_ are essentially the same as for gA but with a shorter time constant and a lower *pH*_IL_ plateau value. This is likely due to the fact that *P*_ALM_>*P*_gA_: the higher membrane permeability of ALM SLBs favours H^+^ transfer (|*j*_H+_|) over H^+^ reduction at the Pd/PdH_*x*_ interface (|*i*_H+_|). In summary, the model developed with our bioprotonic devices provides information on the H^+^ permeability of biomimetic membranes. It should prove valuable to characterize the transport behaviour of other ion channels or H^+^ pumps in biotic–abiotic devices.

## Discussion

In this communication, we demonstrate a biotic–abiotic bioprotonic device with proton (H^+^) conducting Pd/PdH_*x*_ contacts that measures and controls the flow of H^+^ across a lipid bilayer with the ion channels gramicidin A (gA) and alamethicin (ALM). We use gA to increase membrane permeability and control the H^+^ flow across the lipid bilayer with a voltage applied on the Pd/PdH_*x*_ contact. We also demonstrate bidirectional voltage gated switching of the H^+^ flow across the SLB with ALM. Finally, we model H^+^ transport in this system using the Goldman solution and the Nernst equation and use the model to derive the permeability parameters of the lipid bilayer with integrated gA and ALM ion channels and to predict change in pH of the solution at the lipid bilayer/Pd contact interface. Our results indicate that ion channels increase SLB permeability by three orders of magnitude. This is the first time that H^+^ conducting channels have been integrated with Pd/PdH_*x*_ H^+^-conducting contacts and that the H^+^ current flowing through these channels has been directly measured and controlled. Future integration of more complex proteins may require Pd contact functionalization with a self-assembled monolayer^63^ or cushioning lipopolymer^64^,^65^,^66^ to passivate the Pd surface and overcome protein denaturation^66^. To this end, we demonstrate that passivation of the Pd contact with self-assembled 3-aminopropyl-triethoxy-silane^63^ retains gramicidin A device functionality (Supplementary Figs 7–9). On the other hand, passivating the Pd contact with poly (ethylene glycol)^67^,^68^ insulates the Pd surface and hampers H^+^ transfer at the Pd contact solution interface. Alternatively, larger proteins such as *H. turkmenica* detarhodhopsin can be engineered to bind to the Pd contact surface without experiencing denaturation by making use of fused Pd-binding peptides^69^. This work opens the door to integrating more complex active H^+^ channels at the Pd contact interface to produce biotic–abiotic devices with increased functionality.

## Methods

### Materials

1,2-dioleoyl-sn-glycerol-3-phosphocholine (DOPC; Sigma-Aldrich), *Bacillus brevis* Gramicidin A (90% purity; BioChemika), *Trichoderma viride* Alamethicin (90% purity; Sigma-Aldrich) were used as received. The heterofunctional PEG, 1,2-distearoyl-snglycero-3-phosphoethanolamine-N-poly (ethylene glycol)-2000-N- (3-(2-(pyridyldithio) propionate) (DSPE-PEG-PDP) and lipids, L-R-phosphatidylcholine from egg (egg-PC), were purchased from Avanti Polar Lipids (Alabaster, AL). 3-aminopropyl-triethoxy-silane was purchased from, Sigma-Aldrich. Potassium ferrocyanide (K_4_(Fe(CN)_6_), 99.9% purity) K_2_HPO_4_, KH_2_PO_4_, KCl, HCl and KOH (all with 99.9% purity) were purchased from Sigma-Aldrich. K-PBS buffer consisted of 100 mM KCl in 5 mM potassium phosphate (K_2_HPO_4_/KH_2_PO_4_ at a molar ratio of 3:2), pH=7.0. Buffer solutions were prepared with degassed deionized water (Millipore) and the pH was adjusted to 7.0 with HCl or KOH if necessary. The reference and counter electrode (1-mm diameter 3 M KCl Ag/AgCl) was from Warner Instruments. Silicon wafers (100), 4-in. diameter were acquired from International Wafer Service. Ethanol, (200 proof), acetone and chloroform had 99.9% purity.

### Device fabrication and characterization

Bioprotonic devices were fabricated with conventional soft- and photo-lithography on a 100 nm thick layer of silicon dioxide. Each device has a microfluidic SU-8 channel of 4 μm thick. The Pd contacts have varied contact area of 10 μm^2^ (2 × 5 μm), 20 μm^2^ (2 × 10 μm), 40 μm^2^ (2 × 20 μm), 80 μm^2^ (2 × 40 μm), 100 μm^2^ (2 × 50 μm), 120 μm^2^ (2 × 60 μm), 140 μm^2^ (2 × 70 μm), 160 μm^2^ (2 × 80 μm), 180 μm^2^ (2 × 90 μm) and 200 μm^2^ (2 × 100 μm) with a thickness of 50 nm. Pd is deposited on top of 5 nm Cr adhesion layer. Each device has an electrical probe pad out of the microfluidic channel that provides the electrical contact. The microfluidic channel confines the flow of liquid to the top of the Pd contact, while a polydimethylsiloxane well on top of the channel provides space to insert the counter electrode (Supplementary Fig. 1).

### SLB formation and characterization

DOPC vesicles (*d*_avg_=100±23 nm, *n*=3) were prepared by tip sonication of a solution of 0.5 mg ml^−1^ DOPC, in K-PBS buffer solution (pH=7.0). Dynamic light scattering was used to characterize vesicle size. Before deposition, the Pd surface was hydrophilized by oxygen plasma^70^. The vesicle solution was dispensed in the microfluidic channel and the device was gently agitated for 12 h at 100% relative humidity to ensure vesicle fusion, followed by rinsing with K-PBS buffer solution to wash away unfused DOPC vesicles. The resulting bilayers were characterized by AFM, with rupture depth measurements showing a thickness of ca. 4.8±0.7 nm (Supplementary Fig. 2 and Supplementary Note 1)^46^. Home-built systems were used to conduct cyclic voltammetry experiments in the presence of a redox probe and *I*–*V* measurements in the presence of buffer solution. Throughout this process, the pH was monitored using a calibrated pH metre.

### Ion channel incorporation into supported lipid bilayers

Gramicidin A (5 mg ml^−1^) in 200-proof ethanol was mixed with DOPC in chloroform followed by solvent evaporation with N_2_ the rehydration with aqueous buffer solution. DOPC/gramicidin vesicles at a molar ratio of 200:1 were formed by tip sonication and vesicle size determined by dynamic light scattering (*d*_avg_=105±26 nm, *n*=3). These vesicles were then fused onto Pd microfluidic devices. ALM was incorporated into bilayers by incubating 5 mg ml^−1^ solution of ALM peptide for 30 min on freshly deposited Pd-bilayer structures followed by rinsing with K-PBS buffer solution.

### Electrical measurements

All electrical measurements were performed using a Signatone S probe station with a custom built environmental chamber. The probe station was connected to an Agilent 4155C semiconductor parameter analyser.

### Simulations

Iterative simulations were executed in the MATLAB software package. Pd contact Tafel parameters *i*_0_ and *α* were measured with low-voltage *I*–*V* sweeps at a scan rate of 5 mV s^−1^ (Supplementary Fig. 10). The permeability of the three membranes were estimated using a composite model of the permeability of individual cationic channels and the permeability of the DOPC matrix, normalized to their respective occupied area. The length of the isolation layer (ca. 1 nm) was estimated using AFM force versus displacement measurements.

### Code availability

MATLAB code for simulations is available at https://dx.doi.org/10.6084/m9.figshare.3509846.v1.

### Data availability

The data that support the findings of this study are available from the corresponding author on request.

## Additional information

**How to cite this article:** Hemmatian, Z. *et al*. Electronic control of H^+^ current in a bioprotonic device with Gramicidin A and Alamethicin. *Nat. Commun.* **7,** 12981 doi: 10.1038/ncomms12981 (2016).

## Supplementary Material

Supplementary Information Supplementary Figures 1-10, Supplementary Table 1, Supplementary Notes 1-2, Supplementary References

## References

[b1] Jonsson A. . Therapy using implanted organic bioelectronics. Sci. Adv. 1, e1500039 (2015). 26601181 10.1126/sciadv.1500039 PMC4640645

[b2] Pang C. . Highly skin-conformal microhairy sensor for pulse signal amplification. Adv. Mater. 27, 634–640 (2015). 25358966 10.1002/adma.201403807

[b3] Stavrinidou E. . Electronic plants. Sci. Adv. 1, e1501136 (2015). 26702448 10.1126/sciadv.1501136 PMC4681328

[b4] Kim Y. J., Chun S.-E., Whitacre J. & Bettinger C. J. Self-deployable current sources fabricated from edible materials. J. Mater. Chem. B 1, 3781–3788 (2013). 10.1039/c3tb20183j 32261130

[b5] Malliaras G. & Abidian M. R. Organic bioelectronic materials and devices. Adv. Mater. 27, 7492–7492 (2015). 26636956 10.1002/adma.201504783 PMC4682871

[b6] Noy A. Mimicking biology with nanomaterials: carbon nanotube porins in lipid membranes. Biophys. J. 108, 443a (2015).

[b7] Shen Y.-x., Saboe P. O., Sines I. T., Erbakan M. & Kumar M. Biomimetic membranes: a review. J. Membr. Sci. 454, 359–381 (2014).

[b8] Cristof Grewer A. G., Thomas M. & Klaus F. Electrophysiological characterization of membrane transport proteins. Annu. Rev. Biophys. 42, 95–120 (2013). 23451896 10.1146/annurev-biophys-083012-130312

[b9] Watanabe R. . Arrayed lipid bilayer chambers allow single-molecule analysis of membrane transporter activity. Nat. Commun. 5, 4519 (2014). 25058452 10.1038/ncomms5519 PMC4124872

[b10] Oscar Gutierrez-Sanz C. T. . Induction of a proton gradient across a gold-supported biomimetic membrane by electroenzymatic H_2_ oxidation. Angew. Commun. 54, 2684–2687 (2015). 10.1002/anie.201411182 25600156

[b11] Joanna Juhaniewicz S. S. Atomic force microscopy and electrochemical studies of melittin action on lipid bilayers supported on gold electrodes. Electrochim. Acta 162, 63–61 (2015).

[b12] Jahnke J. P., Bazan G. C. & Sumner J. J. Effect of modified phospholipid bilayers on electrochemical activity of a membrane-spanning conjugated oligoelectrolyte. Langmuir 31, 11613–11620 (2015). 26422050 10.1021/acs.langmuir.5b03093

[b13] Tse E. C. . Anion transport through lipids in a hybrid bilayer membrane. Anal. Chem. 87, 2403–2409 (2015). 25597547 10.1021/ac5043544

[b14] Buck R. P. Kinetics of bulk and interfacial ionic motion: microscopic basis and limits for the Nernst-Planck equation applied to membrane systems. J. Membr. Sci. 17, 1–62 (1984).

[b15] Robertson J. W. Modeling ion transport in tethered bilayer lipid membranes. 1. Passive ion permeation. J. Phys. Chem. B 112, 10475–10482 (2008). 18680332 10.1021/jp800162d

[b16] DeCoursey T. E. The voltage-gated proton channel: a riddle, wrapped in a mystery, inside an enigma. Biochemistry 54, 3250–3268 (2015). 25964989 10.1021/acs.biochem.5b00353 PMC4736506

[b17] Morowitz H. J. Proton semiconductors and energy transduction in biological systems. Am. J. Physiol.-Regul. Integr. Comp. Physiol. 235, R99–R114 (1978). 10.1152/ajpregu.1978.235.3.R99 696856

[b18] Lanyi J. K. Bacteriorhodopsin. Annu. Rev. Physiol. 66, 665–688 (2004). 14977418 10.1146/annurev.physiol.66.032102.150049

[b19] Smith S. M. . Voltage-gated proton channel in a dinoflagellate. Proc. Natl Acad. Sci. USA 108, 18162–18167 (2011). 22006335 10.1073/pnas.1115405108 PMC3207696

[b20] Walz D. & Caplan S. R. Bacterial flagellar motor and H^+^/ATP synthase: two proton-driven rotary molecular devices with different functions. Bioelectrochemistry 55, 89–92 (2002). 11786348 10.1016/s1567-5394(01)00162-1

[b21] Capasso M., DeCoursey T. E. & Dyer M. J. pH regulation and beyond: unanticipated functions for the voltage-gated proton channel, HVCN1. Trends Cell Biol. 21, 20–28 (2011). 20961760 10.1016/j.tcb.2010.09.006 PMC3014425

[b22] Busath D. & Szabo G. Gramicidin forms multi-state rectifying channels. Nature 294, 371–373 (1981). 6171731 10.1038/294371a0

[b23] Ajo-Franklin C. M. & Noy A. Crossing over: nanostructures that move electrons and ions across cellular membranes. Adv. Mater. 27, 5797–5804 (2015). 25914282 10.1002/adma.201500344

[b24] Huang S.-C. J. . Carbon nanotube transistor controlled by a biological ion pump gate. Nano Lett. 10, 1812–1816 (2010). 20426455 10.1021/nl100499x

[b25] Misra N. . Bioelectronic silicon nanowire devices using functional membrane proteins. Proc. Natl Acad. Sci. 106, 13780–13784 (2009). 19667177 10.1073/pnas.0904850106 PMC2728971

[b26] Angione M. D. . Interfacial electronic effects in functional biolayers integrated into organic field-effect transistors. Proc. Natl Acad. Sci. USA 109, 6429–6434 (2012). 22493224 10.1073/pnas.1200549109 PMC3340085

[b27] Johnson N., Kim Y. J., Dingh H., LeDuc P. & Bettinger C. Bio-Inspired pH responsive hydrogels for programmed activation of electrochemical storage systems in biology. Biophys. J. 108, 485a (2015).

[b28] Mitraka E. . Solution processed liquid metal-conducting polymer hybrid thin films as electrochemical pH-threshold indicators. J. Mater. Chem. C 3, 7604–7611 (2015).

[b29] Owens R. M. & Malliaras G. G. Organic electronics at the interface with biology. MRS Bull. 35, 449–456 (2010).

[b30] Tybrandt K., Larsson K. C., Richter-Dahlfors A. & Berggren M. Ion bipolar junction transistors. Proc. Natl Acad. Sci. 107, 9929–9932 (2010). 20479274 10.1073/pnas.0913911107 PMC2890459

[b31] Williamson A. . Epilepsy treatment: controlling epileptiform activity with organic electronic ion pumps. Adv. Mater. 27, 3097–3097 (2015). 10.1002/adma.201500482 25866154

[b32] Tybrandt K., Forchheimer R. & Berggren M. Logic gates based on ion transistors. Nat. Commun. 3, 871 (2012). 22643898 10.1038/ncomms1869

[b33] Deng Y. . H^+^-type and OH^−^-type biological protonic semiconductors and complementary devices. Sci. Rep. 3, 12118 (2013). 10.1038/srep02481 PMC3789148 24089083

[b34] Miyake T. & Rolandi M. Grotthuss mechanisms: from proton transport in proton wires to bioprotonic devices. J. Phys.: Condens. Matter 28, 023001 (2015). 26657711 10.1088/0953-8984/28/2/023001

[b35] Zhong C. . A polysaccharide bioprotonic field-effect transistor. Nat. Commun. 2, 476 (2011). 21934660 10.1038/ncomms1489

[b36] Josberger E. E., Deng Y., Sun W., Kautz R. & Rolandi M. Two-terminal protonic devices with synaptic-like short-term depression and device memory. Adv. Mater. 26, 4986–4990 (2014). 24789251 10.1002/adma.201400320

[b37] Miyake T., Josberger E. E., Keene S., Deng Y. & Rolandi M. An enzyme logic bioprotonic transducer. APL Mater. 3, 014906 (2015).

[b38] Ordinario D. D. . Bulk protonic conductivity in a cephalopod structural protein. Nat. Chem. 6, 596–602 (2014). 24950329 10.1038/nchem.1960

[b39] Rolandi M. Bioelectronics: a positive future for squid proteins. Nat. Chem. 6, 563–564 (2014). 24950323 10.1038/nchem.1980

[b40] Ordinario D. D., Phan L., Jocson J.-M., Nguyen T. & Gorodetsky A. A. Protonic transistors from thin reflectin films. APL Mater. 3, 014907 (2015).

[b41] Glasser L. Proton conduction and injection in solids. Chem. Rev. 75, 21–65 (1975).

[b42] Kowal J. Ł. . Functional surface engineering by nucleotide-modulated potassium channel insertion into polymer membranes attached to solid supports. Biomaterials 35, 7286–7294 (2014). 24912817 10.1016/j.biomaterials.2014.05.043

[b43] Aghdaei S., Sandison M. E., Zagnoni M., Green N. G. & Morgan H. Formation of artificial lipid bilayers using droplet dielectrophoresis. Lab Chip 8, 1617–1620 (2008). 18813381 10.1039/b807374k

[b44] Hemmatian Z. . Taking electrons out of bioelectronics: bioprotonic memories, transistors, and enzyme logic. J. Mater. Chem. C 3, 6407–6412 (2015).

[b45] Stroumpoulis D., Parra A. & Tirrell M. A kinetic study of vesicle fusion on silicon dioxide surfaces by ellipsometry. Aiche J. 52, 2931–2937 (2006).

[b46] Attwood S. J., Choi Y. & Leonenko Z. Preparation of DOPC and DPPC supported planar lipid bilayers for atomic force microscopy and atomic force spectroscopy. Int. J. Mol. Sci. 14, 3514–3539 (2013). 23389046 10.3390/ijms14023514 PMC3588056

[b47] Huang S.-C. J. . Formation, stability, and mobility of one-dimensional lipid bilayers on polysilicon nanowires. Nano Lett. 7, 3355–3359 (2007). 17900161 10.1021/nl071641w

[b48] Leonenko Z., Finot E., Ma H., Dahms T. & Cramb D. Investigation of temperature-induced phase transitions in DOPC and DPPC phospholipid bilayers using temperature-controlled scanning force microscopy. Biophys. J. 86, 3783–3793 (2004). 15189874 10.1529/biophysj.103.036681 PMC1304279

[b49] Boxer S. G. Molecular transport and organization in supported lipid membranes. Curr. Opin. Chem. Biol. 4, 704–709 (2000). 11102877 10.1016/s1367-5931(00)00139-3

[b50] Richter R. P. & Brisson A. R. Following the formation of supported lipid bilayers on mica: a study combining AFM, QCM-D, and ellipsometry. Biophys. J. 88, 3422–3433 (2005). 15731391 10.1529/biophysj.104.053728 PMC1305489

[b51] Tamm L. K. & McConnell H. M. Supported phospholipid bilayers. Biophys. J. 47, 105 (1985). 3978184 10.1016/S0006-3495(85)83882-0 PMC1435076

[b52] Johnson S. . Structure of an adsorbed dimyristoylphosphatidylcholine bilayer measured with specular reflection of neutrons. Biophys. J. 59, 289 (1991). 2009353 10.1016/S0006-3495(91)82222-6 PMC1281145

[b53] Woolley G. A. & Wallace B. Model ion channels: gramicidin and alamethicin. J. Membr. Biol. 129, 109–136 (1992). 1279177 10.1007/BF00219508

[b54] Boheim G., Hanke W. & Jung G. Alamethicin pore formation: voltage-dependent flip-flop of α-helix dipoles. Biophys. Struct. Mech. 9, 181–191 (1983).

[b55] Cafiso D. Alamethicin: a peptide model for voltage gating and protein-membrane interactions. Annu. Rev. Biophys. Biomol. Struct. 23, 141–165 (1994). 7522664 10.1146/annurev.bb.23.060194.001041

[b56] Mottamal M. & Lazaridis T. Voltage-dependent energetics of alamethicin monomers in the membrane. Biophys. Chem. 122, 50–57 (2006). 16542770 10.1016/j.bpc.2006.02.005

[b57] Bechinger B. Structure and functions of channel-forming peptides: magainins, cecropins, melittin and alamethicin. J. Membr. Biol. 156, 197–211 (1997). 9096062 10.1007/s002329900201

[b58] Mathew M. K., Nagaraj R. & Balaram P. Membrane channel-forming polypeptides. Aqueous phase aggregation and membrane-modifying activity of synthetic fluorescent alamethicin fragments. J. Biol. Chem. 257, 2170–2176 (1982). 6277888

[b59] Dencher N. A. H. J., Büldt G., Höltje H. D. & Höltje M. in *Membrane Proteins: Structures Interactions and Models*, 69 (eds Pullman, A. .) (Kluwer Academic Publisher, 1992).

[b60] Hodgkin A. L. & Katz B. The effect of sodium ions on the electrical activity of the giant axon of the squid. J. Physiol. 108, 37–77 (1949). 18128147 10.1113/jphysiol.1949.sp004310 PMC1392331

[b61] Kulikovsky A. Analytical model of the anode side of DMFC: the effect of non-Tafel kinetics on cell performance. Electrochem. Commun. 5, 530–538 (2003).

[b62] Potvin P. G. Modelling complex solution equilibria. I. Fast, worry-free least-squares refinement of equilibrium constants. Can. J. Chem. 68, 2198–2207 (1990).

[b63] Leonenko Z., Carnini A. & Cramb D. Supported planar bilayer formation by vesicle fusion: the interaction of phospholipid vesicles with surfaces and the effect of gramicidin on bilayer properties using atomic force microscopy. Biochim. Biophys. Acta (BBA)-Biomembr. 1509, 131–147 (2000). 10.1016/s0005-2736(00)00288-1 11118525

[b64] Diaz A. J., Albertorio F., Daniel S. & Cremer P. S. Double cushions preserve transmembrane protein mobility in supported bilayer systems. Langmuir 24, 6820–6826 (2008). 18510376 10.1021/la800018d PMC3475160

[b65] Wong J. Y. . Polymer-cushioned bilayers. I. A structural study of various preparation methods using neutron reflectometry. Biophys. J. 77, 1445–1457 (1999). 10465755 10.1016/S0006-3495(99)76992-4 PMC1300432

[b66] Castellana E. T. & Cremer P. S. Solid supported lipid bilayers: from biophysical studies to sensor design. Surface Sci. Rep. 61, 429–444 (2006). 10.1016/j.surfrep.2006.06.001 PMC7114318 32287559

[b67] Jin J. . A biomimetic composite from solution self-assembly of chitin nanofibers in a silk fibroin matrix. Adv. Mater. 25, 4482–4487 (2013). 23788326 10.1002/adma.201301429

[b68] Munro J. C. & Frank C. W. *In situ* formation and characterization of poly (ethylene glycol)-supported lipid bilayers on gold surfaces. Langmuir 20, 10567–10575 (2004). 15544386 10.1021/la048378o

[b69] Soto-Rodríguez J., Hemmatian Z., Josberger E. E., Rolandi M. & Baneyx F. A palladium-binding deltarhodopsin for light-activated conversion of protonic to electronic currents. Adv. Mater. 28, 6581–6585 (2016). 27185384 10.1002/adma.201600222

[b70] Weber M. J. . Atomic layer deposition of high-purity palladium films from Pd (hfac)_2_ and H_2_ and O_2_ plasmas. J. Phys. Chem. C 118, 8702–8711 (2014).

