# Supplementary material for: Electronic control of H+ current in a bioprotonic device with Gramicidin A and Alamethicin
Source: Nat Commun. 2016 Oct 7;7:12981. doi: 10.1038/ncomms12981 (PMC5059763; doi:10.1038/ncomms12981)
Supplement: Supplementary Information — Supplementary Figures 1-10, Supplementary Table 1, Supplementary Notes 1-2, Supplementary References [file ncomms12981-s1.pdf]

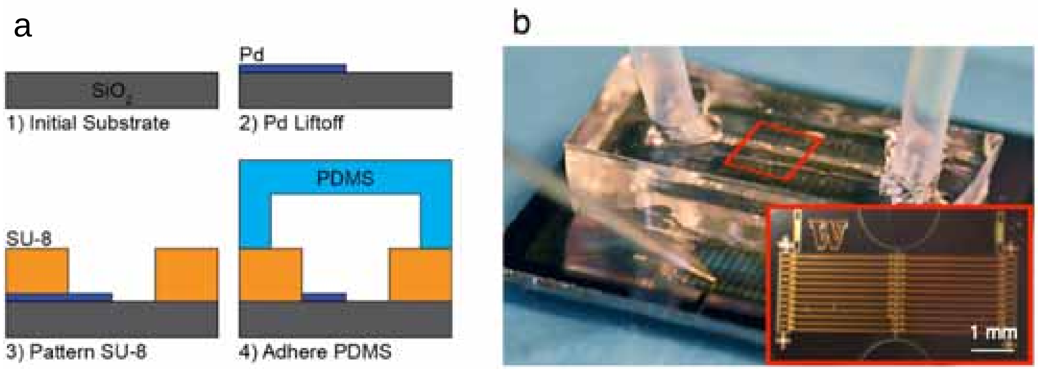

Supplementary Figure 1 a) Scheme of microfluidic device fabrication by photo and soft lithography, (a1, a2) 50nm Pd evaporated on Si wafer with 100 nm SiO_2 insulating layer and 5nm Cr as an adhesion layer (a3) A layer of SU-8 photoresist with the thickness of 4μm forms a microfluidic channel. (a4) The device is sealed with a PDMS layer to inlet and outlet port room to insert a Ag/ AgCl electrode (b) Photograph of the bioprotonic microfluidic device; described above, insert is top view.

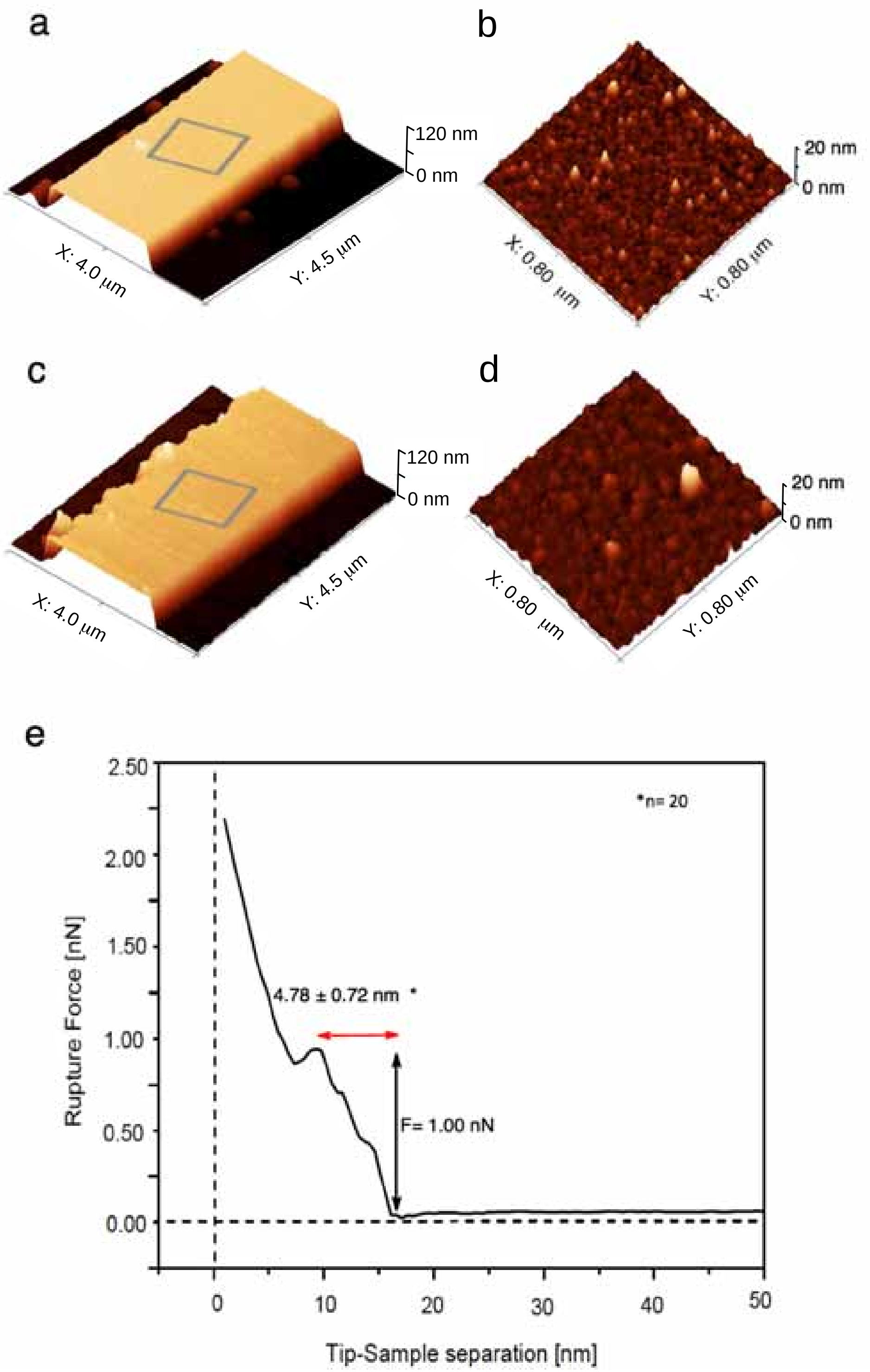

Supplementary Figure 2 AFM characterization of SLB formation on the Pd contact, (a) Tapping mode AFM image of a naked Pd contact after O_2 plasma cleaning (b) Zoom in of a naked Pd contact shows an almost flat substrate with a roughness of ca. 0.5 ± 0.0 nm (c) AFM image of Pd contact covered with SLB, (d) Zoom in of a Pd covered with SLB shows increase in roughness of ca. 0.7 ± 0.1 nm (e) A representative force-distance curve for rupture of SLB in liquid following the protocol from literature.[5] The rupture occurs at F= 1 nN, F is the force between the AFM tip and the sample surface with repulsive force oriented upwards on the y- axis. There is attraction right at a distance at 16.2 nm from the Pd surface by the small dip in the graph, indicating the tip first senses the bilayer. The rupture depth occurs at 4.8 ± 0.7 nm.

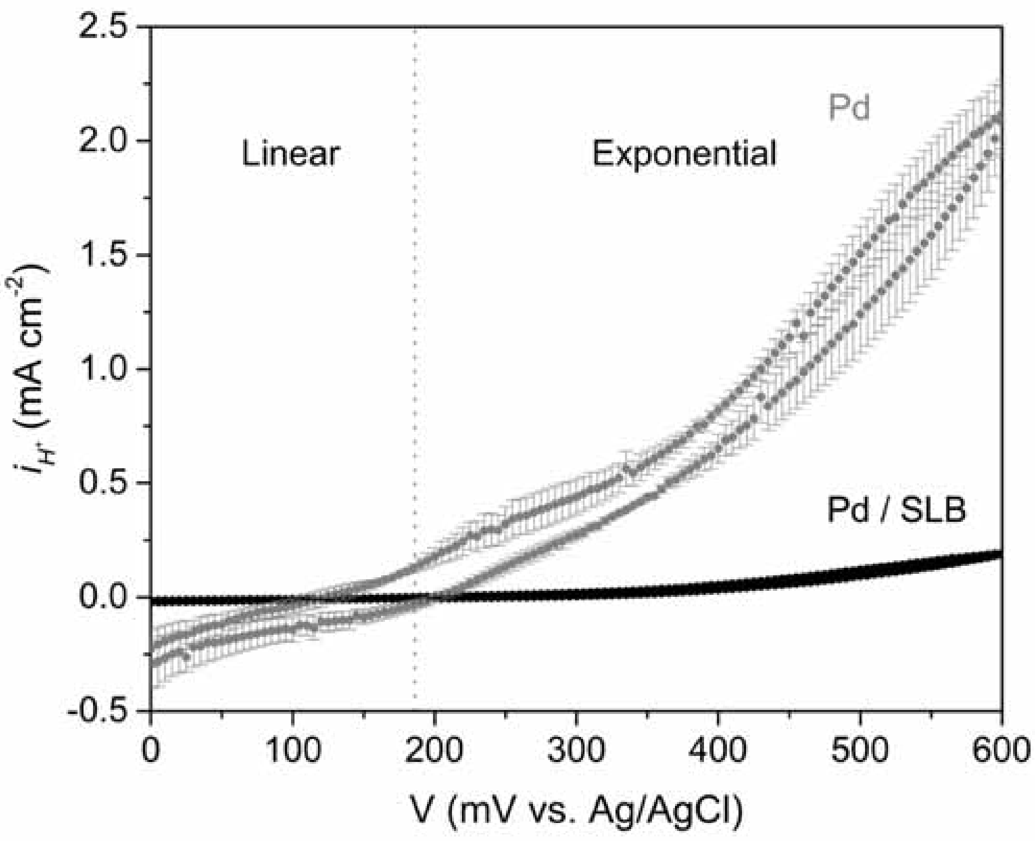

Supplementary Figure 3 I-V sweep for SLB formation, the electrical characteristics and integrity of the SLB are characterized with I-V measurements and ferrocyanide ($[Fe(CN\text{-})_6]^{4-}$) is used as a redox probe in a two terminal configuration where Ag/AgCl is used as a reference and counter electrode. i_{H+} max (V= 600mV) for the Pd contact covered with the SLB (black trace) is ca.11 times smaller than for the naked Pd contact (gray trace). (The data is collected from 3 different devices with the different dimensions: Pd/ SLB: 3 different devices of 2 μm × 50 μm, Naked Pd: 2 different devices of 2 μm × 50 μm and 1 device of 2 μm × 20 μm. The error bars are the root mean square (RMS) of the displacement of the data from the average value)

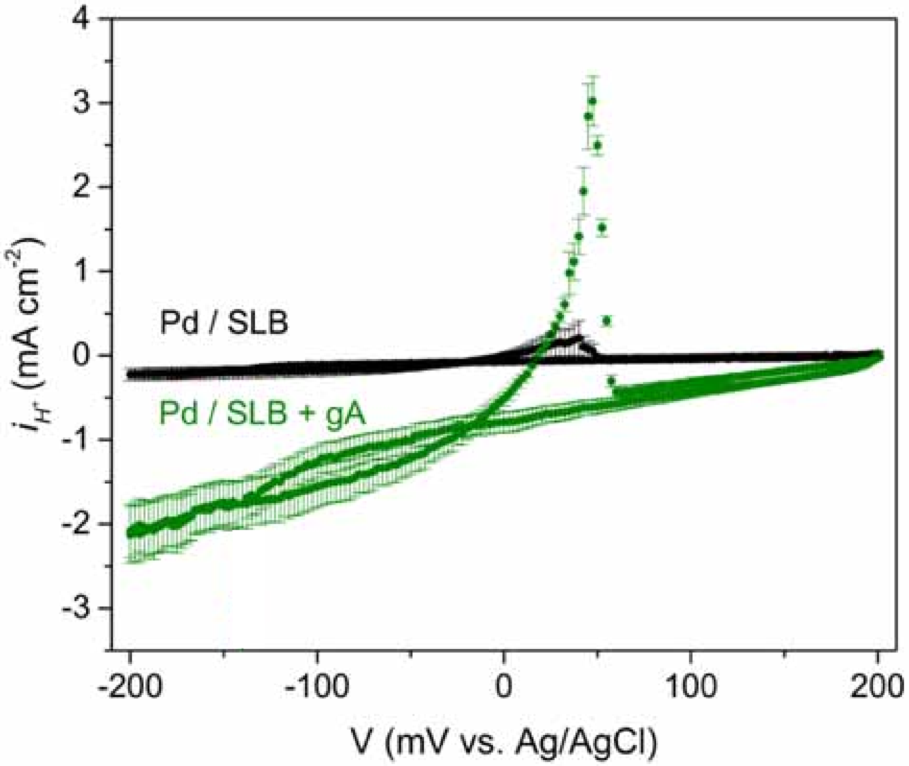

Supplementary Figure 4 I-V sweep of gA device, Pd / SLB has low $i_{H+} > -0.225$ mA cm^{-2} across the entire voltage range, with a small oxidation peak with i_{H+} max of ca. 0.2 mA cm^{-2}, confirming the high polarization resistance of the SLB (black trace). Bioprotonic devices with integrated gA have higher i_{H+} max of ca. 3 mA cm^{-2}, with a large PdH_x oxidation peak at 50 mV [3], indicating H^+ across the SLB membrane (green trace). (The data is collected from 3 different devices with the different dimensions: Pd/ SLB: 3 different devices of 2 μm × 50 μm, Pd/ SLB + gA: 2 μm × 20 μm, 2 μm × 50 μm, 2 μm × 70 μm. The error bars are the root mean square (RMS) of the displacement of the data from the average value).

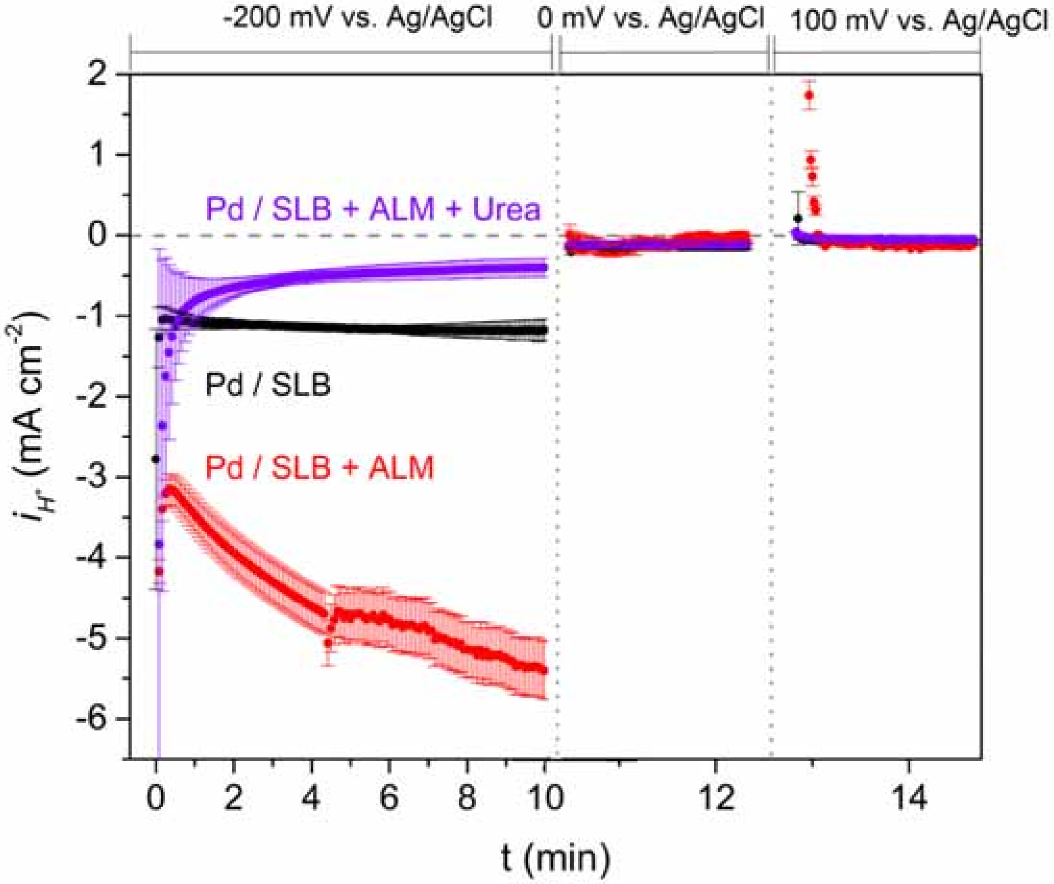

Supplementary Figure 5 I-t plot of ALM disruption by introducing urea, Urea disrupts the ALM molecules, removing the ALM channels from the SLB [2]. At V= -200 mV, i_{H+} = -5.5 mA cm^{-2} for SLB+ ALM (red trace) while the channel is open and at V= 100 mV, i_{H+} = 1.9 mA cm^{-2} for SLB+ ALM (red trace). Addition of 40 mM urea to solution disrupts the aggregation of individual ALM monomers, it brings the corresponding i_{H+} to -0.4 mA cm^{-2} at V= -200 mV (purple trace) and i_{H+} = 0 mA cm^{-2} by the follow up at V= 100mV (blue trace). (The data is collected from 3 different devices with the different dimensions, Pd/ SLB: 3 different devices of 2 μm × 50 μm, Pd/ SLB + ALM: 2 μm × 20 μm, 2 μm × 40 μm, 2 μm × 50 μm, Pd/ SLB + ALM + Urea: 2 μm × 20 μm, 2 μm × 40 μm, 2 μm × 50 μm. The error bars are the root mean square (RMS) of the displacement of the data from the average value)

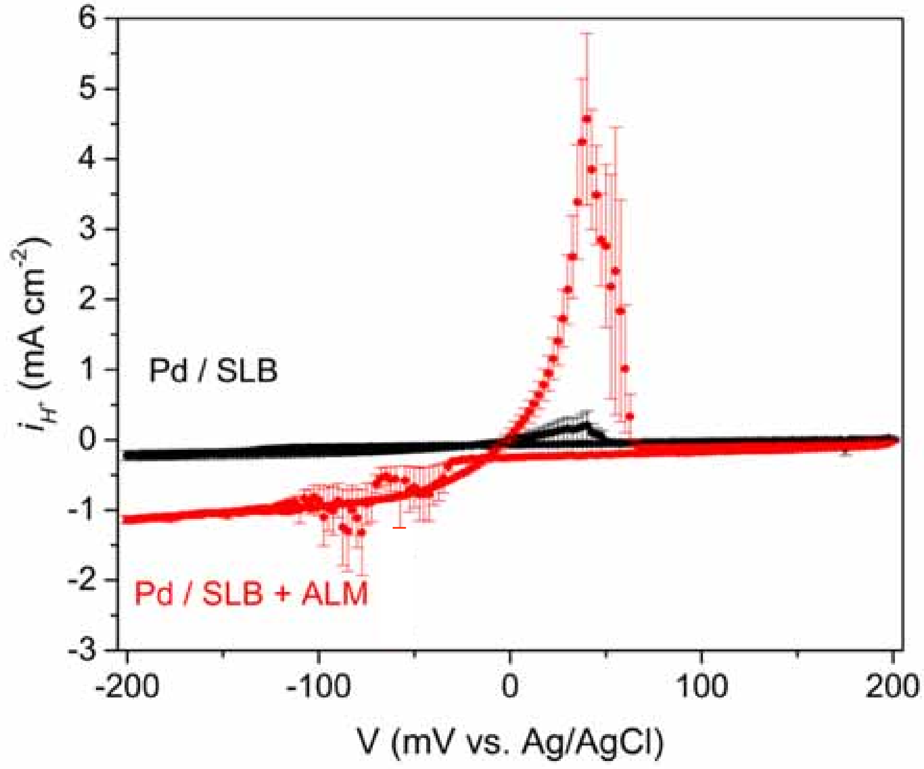

Supplementary Figure 6 I-V Sweep of ALM voltage-gated device, Pd / SLB has a low i_{H+} > -0.225 mA cm^{-2} across the entire voltage range, with a small oxidation peak with i_{H+} max of ca. 0.2 mA cm^{-2}, confirming the high polarization resistance of the SLB (black trace). Bioprotonic devices with integrated with ALM have similar polarization resistances to SLB at low V due to the high impedance across the SLB membrane when ALM channels are closed. Additionally, we observe a large PdH_x oxidation peak with a max i_{H+} of ca. 45 mA cm^{-2} at V= 50 mV [3], indicating H^+ flow across the SLB (red trace). (The data is collected from 3 different devices with the different dimensions, Pd/ SLB: 3 different devices of 2 μm × 50 μm, Pd/ SLB + ALM: 2 μm × 20 μm, 2 μm × 40 μm, 2 μm × 50 μm. The error bars are the root mean square (RMS) of the displacement of the data from the average value).

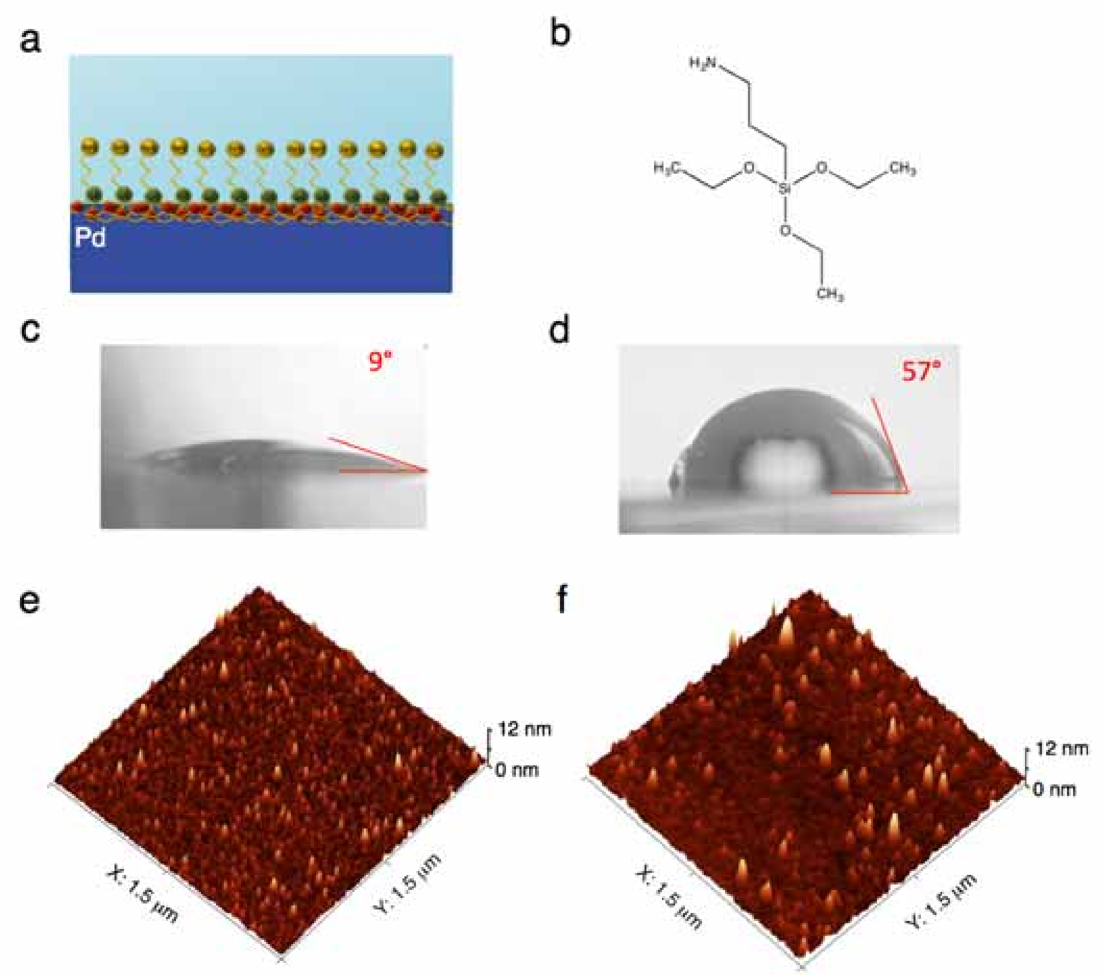

Supplementary Figure 7 Surface tension and AFM characterization of APTES self-assembly on Pd contact, (a) Scheme of the Pd device covered with APTES [1](b) Molecular structure of APTES (c) Surface tension measurements of a naked Pd contact after O_2 plasma cleaning of ca. 9° (d) Surface tension measurements of Pd contact covered by APTES shows increase on the contact angle to ca. 57° (e) AFM image in tapping mode of a naked Pd contact after plasma cleaning, shows an almost flat substrate with a roughness of ca. 0.6 ± 0.1 nm (f) AFM image in tapping mode of the Pd contact covered by ATPES shows a decrease in roughness to ca. 0.3 ± 0.1 nm. (n= 3, for the device with dimension of 2 μm × 50 μm).

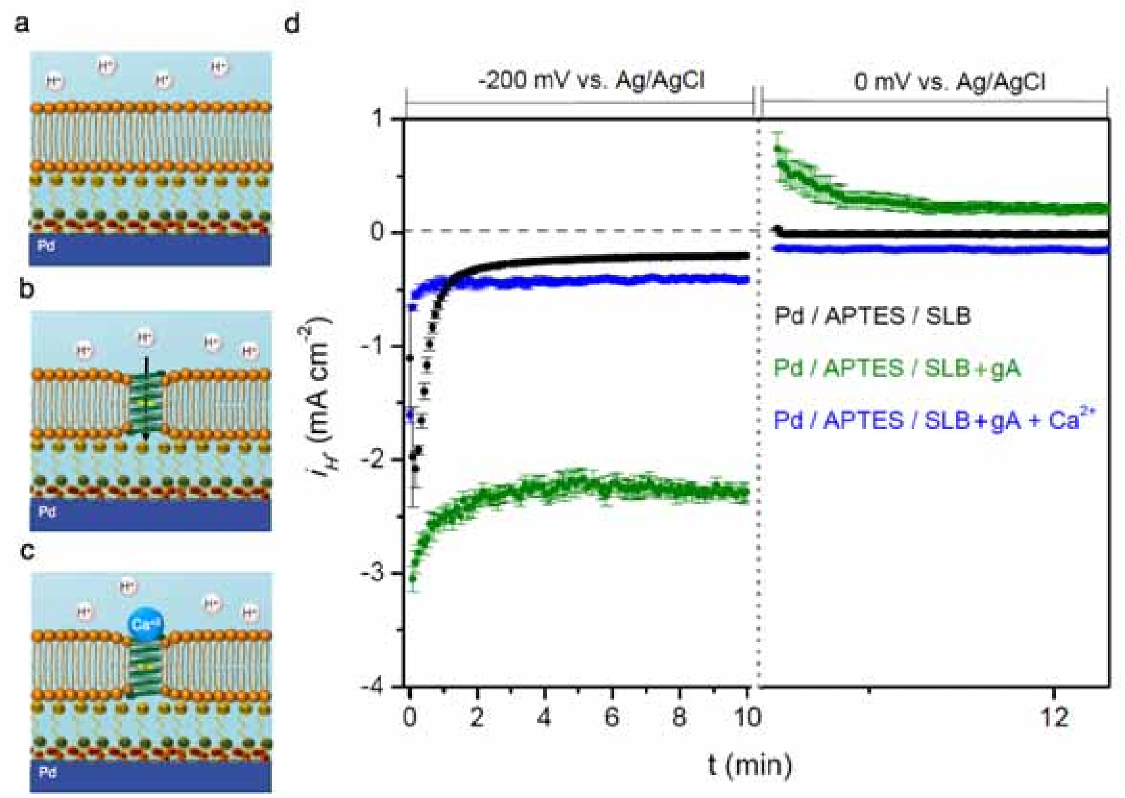

Supplementary Figure 8 (a, b, c) Schematics of bioprotonic gA devices with the self-assembly of APTES. (a) Pd contact coated with APTES / SLB. The APTES / SLB inhibits the flux of H^+ from the bulk solution to the Pd/solution interface. (b) Pd contact with APTES / SLB incorporating gA is semipermeable to H^+, with gA channels facilitating the rapid flow of H^+ to the Pd solution interface (c) Addition of 1 mM Ca^{2+} to the bulk solution, blocks gA and prevents the flow of H^+ to the Pd / APTES/ solution interface (d) i_{H+}-t plot for V = -200 mV and V = 0 mV for APTES / SLB (black trace) and APTES / SLB + gA (green trace) and APTES / SLB+ gA + Ca^{2+} (blue trace) on a Pd contact. (The data is collected from 3 different devices with the different dimensions, Pd/ APTES /SLB: 2 μm × 50 μm, and 2 different device of 2 μm × 85 μm, Pd/ APTES / SLB + gA: 2 μm × 50 μm and 2 different devices of 2 μm × 80 μm, Pd/ APTES/ SLB+ gA+ Ca^{2+} 2 μm × 50 μm and 2 different devices of 2 μm × 80 μm. The error bars are the root mean square (RMS) of the displacement of the data from the average value).

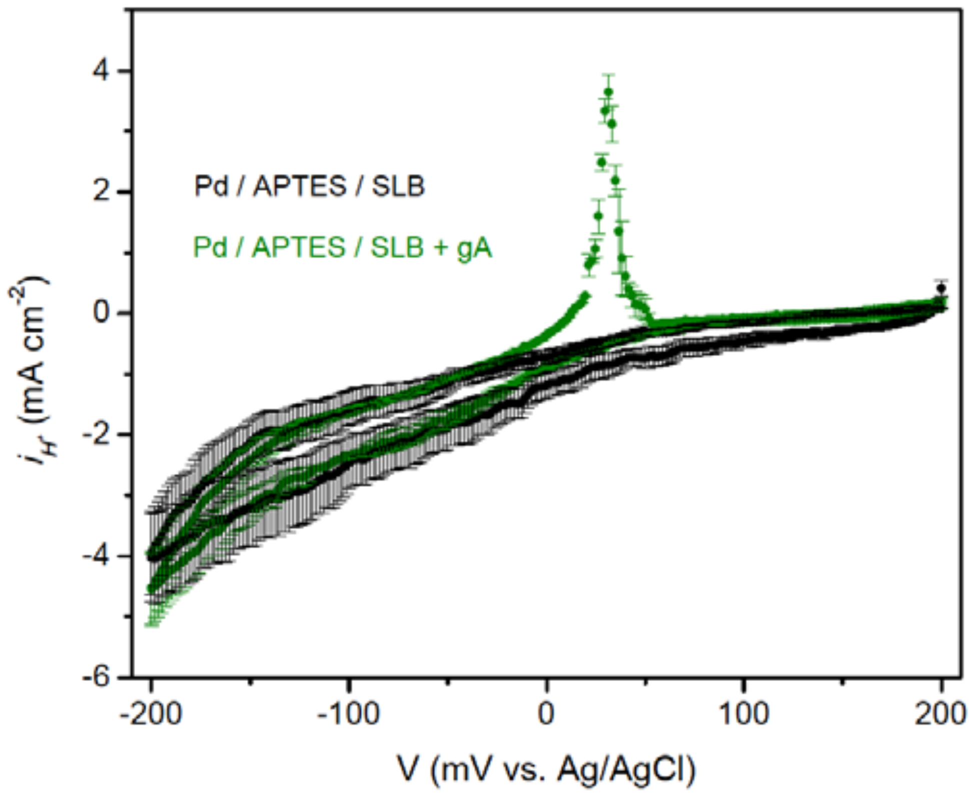

Supplementary Figure 9 I-V sweep of gA device, Pd / APTES / SLB has i_{H+} > -4mA cm^{-2} across the entire voltage range, without oxidation peak, confirming the high polarization resistance of the APTES / SLB (black trace). Bioprotonic devices with integrated gA have higher i_{H+} max of ca. 4 mA cm^{-2}, with a large PdH_x oxidation peak at 45 mV [3], indicating H^+ across the APTES / SLB membrane (green trace). (The data is collected from 3 different devices with the different dimensions: Pd/ SLB: 3 different devices of 2 μm × 85 μm, Pd/ SLB + gA: 2 μm × 40 μm, 2 μm × 50 μm, 2 μm × 70 μm. The error bars are the root mean square (RMS) of the displacement of the data from the average value).

a

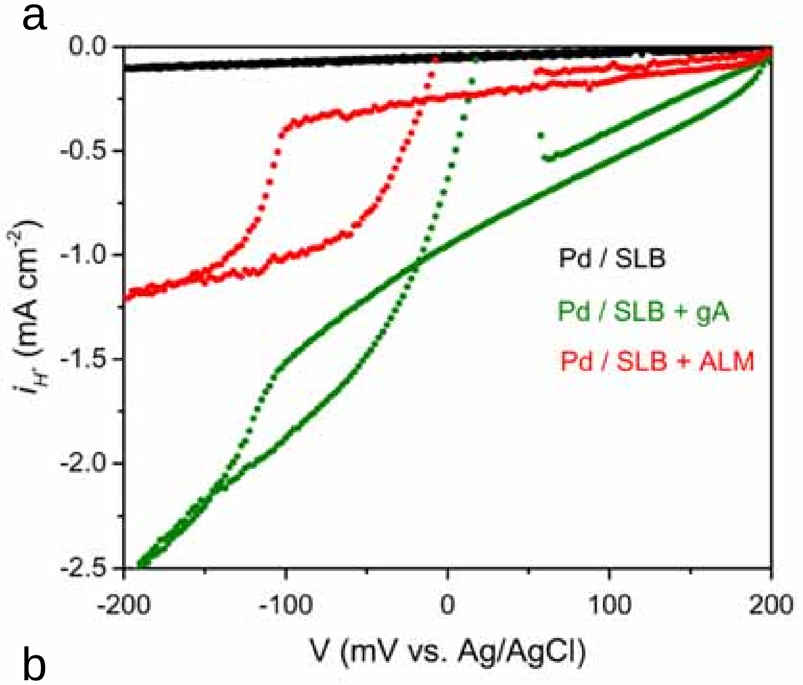

b

	Pd / SLB	Pd / SLB + gA	Pd / SLB + ALM
Slope [mA cm^{-2} V^{-1}]	0.25436	3.91856	0.8907
Intercept [mA cm^{-2}]	0.05852	0.9171	0.24632
i_0 [mA cm^{-2}]	0.00658	0.10136	0.02304

Supplementary Figure 10 (a) I-V sweep of bioprotonic device with electrochemical redox reaction of H^+ for the Pd contact with SLB (black trace), Pd contact with SLB + gA (green trace), and Pd contact with SLB + ALM (red trace). (b) Tafel fitting parameters are applied for the low polarization (linear) regime yielding the H^+ exchange current at the respective electrodes. (The data is collected from the devices with dimension of 2 μm

Parameter	SLB On Pd contact
$\beta_{a\text{ -bare Pd}}$	0.452 ± 0.093
$\beta_{a\text{-SLB}}$	0.254 ± 0.025
K^{0}_{LB} (cm/s)	$(6.82 \pm 2.45) \times 10^{-4}$
$K^{0}_{SLB} / K^{0}_{\text{bare Pd}}$	0.93
$I_{SLB}/I_{\text{bare Pd}}$ (600 mV)	0.12 ± 0.03

Supplementary Table 1 Tafel parameter on SLB formation by I-V characterization, the Tafel electrode parameters extracted from the low polarization and exponential regimes. These values show similar ionic insulation as compared with reported electrodes with integrated SLB.[4]

Exponential

$$\mathrm{Ln}(I) = \ln(FAk^{0}C^{0}) + f\beta(V - V^{0})$$

β_a = anodic transfer coefficient
k^{0} = reaction rate constant
C^{0} = initial concentration
$f = F / RT$
V^{0} = standard reaction potential of redox probe

Linear

$$I = I^{0} f (V - V^{0})$$

I^{0} = exchange current density

Supplementary Note 1

The AFM measurements on SLB formation were performed in liquid using the Bruker liquid cell holder using Bruker's Peak Force method. We used Scan-Asyst fluid cantilevers for all measurements. For topography images, the Bruker's Peak Force method was used with forces always under F = 1 nN, in order to prevent damage to the surface. F is the force between the AFM tip and the sample surface with repulsive force oriented upwards on the y- axis and it is calculated by the AFM software based on the cantilever deflection and cantilever spring constant. Distance is measured by Piezo element in the head. For the force-distance measurements, each data (n= 20, for the device with dimension of 2 μm × 50 μm) was collected at a single point on the film, with forces high enough to penetrate the film.

Supplementary Note 2

Equation 1 to Equation 5:

$$\beta \ = -2.303([H^+\ ]IL \quad + [OH^-]_{IL} + \sum_{j=0}^{n} \frac{C_{IL} K_{a,j} [H^+]_{IL}}{(K_{a,j} + [H^+]_{IL})^2}) \qquad 6$$

Where the ($[OH^-]_{IL}$ is the hydroxide concentration in the IL ($[OH]_{IL}$ = Kw/$[H^+]_{IL}$), C_{IL} is the concentration of the corresponding buffering acid (KH_2PO_4 or H_3PO_4), K_a is the acid dissociation constant of the buffering acid, and β is the buffer capacity.

We use a standard buffer solution of 5mM PBS and 100mM KCl, resulting in the following pH response:

$$[K_2HPO_4] + [KH_2PO_4] + [H_3PO_4] = 5{\times}10^{-3}\ M \qquad (2)$$

$$pH = pK_a + log\frac{[KA]}{[HA]} \qquad (3)$$

Where [HA] and [KA] are the conjugate acid and base concentrations in the isolation layer, respectively. We use equation 2 and equation 3 to solve a system of equations that results in equations to calculate each acid concentration as a function of pH:

$$[KH_2PO_4] = \frac{5*10^{-3}}{10^{pH-6.8} + 10^{2.148-pH} + 1}\ M \qquad (4)$$

$$[H_3PO_4] = \frac{5*10^{pH-5.148}}{10^{pH-6.8} + 10^{2.148-pH} + 1}\ M \qquad (5)$$

Supplementary References

1 Leonenko, Z., Carnini, A. & Cramb, D. Supported planar bilayer formation by vesicle fusion: the interaction of phospholipid vesicles with surfaces and the effect of gramicidin on bilayer properties using atomic force microscopy. Biochimica et Biophysica Acta (BBA)-Biomembranes 1509, 131-147 (2000).

2 Mathew, M. K., Nagaraj, R. & Balaram, P. Membrane channel-forming polypeptides. Aqueous phase aggregation and membrane-modifying activity of synthetic fluorescent alamethicin fragments. Journal of Biological Chemistry 257, 2170-2176 (1982).

3 Chevillot, J.-P., Farcy, J., Hinnen, C. & Rousseau, A. Electrochemical study of hydrogen interaction with palladium and platinum. Journal of Electroanalytical Chemistry and Interfacial Electrochemistry 64, 39-62 (1975).

4 Misra, N. et al. Bioelectronic silicon nanowire devices using functional membrane proteins. Proceedings of the National Academy of Sciences 106, 13780-13784 (2009).

5 Attwood, S. J., Choi, Y. & Leonenko, Z. Preparation of DOPC and DPPC supported planar lipid bilayers for Atomic Force Microscopy and Atomic Force Spectroscopy. International journal of molecular sciences 14, 3514-3539 (2013).

6 The Melanin Binding of Drugs and Its Implications. Drug Metabolism Reviews 15, 1183-1212, doi:10.3109/03602538409033561 (1984).
